# Pharmacokinetics and Biodistribution of Pegylated Methotrexate after IV Administration to Mice

**Published:** 2018

**Authors:** Gholamhossein Yousefi, Alireza Shafaati, Afshin Zarghi, Seyed Mohsen Foroutan

**Affiliations:** a *Department of Pharmaceutics, School of Pharmacy, Shiraz University of Medical Sciences, Shiraz, Iran.*; b *Center for Nanotechnology in Drug Delivery, Shiraz University of Medical Sciences, Shiraz, Iran.*; c *Depratment of Pharmaceutical Chemistry, School of Pharmacy, Shahid Beheshti University of Medical Sciences, Tehran, Iran. *; d *Department of Pharmaceutics, School of Pharmacy & Protein Technology Research Center, Shahid Beheshti University of Medical Sciences,Tehran, Iran.*

**Keywords:** Methotrexate, Pegylation, Pharmacokinetics, Biodistribution, Mice, Stability

## Abstract

The efficacy of methotrexate (MTX) as an antimetabolite chemotherapeutic agent highly depends on its blood circulation half-life. In our previous study, different conjugates of MTX (MTX-PEG) were synthesized, their physicochemical properties were investigated and MTX-PEG5000 was finally selected as optimum drug-conjugate for further investigations. In the current work, first the stability of MTX-PEG5000 was studied at 37 °C and the results indicated its high stability in plasma (T_1/2_ = 144 h) and a relatively rapid degradation in tissue homogenate (T_1/2_ = 24 h). The study of protein binding pointed out that the conjugate was highly protein-bound (95%). The results of pharmacokinetic studies in mice indicated that MTX-PEG5000 had longer plasma distribution and elimination half-lives compared to free MTX (T_1/2__α_ 9.16 min for MTX-PEG5000 versus 2.45 min for MTX and T_1/2__β_ 88.44 for MTX-PEG5000 versus 24.33 min for MTX). Pharmacokinetic parameters also showed higher area under the curve (AUC) of conjugate compared to parent drug (12.33 mg.mL^-1^.min for MTX-PEG5000 versus 2.64 mg.mL^-1^.min for MTX). The biodistribution studies demonstrated that MTX-PEG5000 did not highly accumulate in liver and intestine and had a mild and balanced distribution to other organs. Also, the conjugate was measurable in tissues up to 48 h after injection and was detected in the brain, suggesting the possibility of delivering drug to brain tumors.

## Introduction

Methotrexate (MTX), the 4-amino, 10-methyl analogue of folic acid, is the most widely used antifolate in cancer chemotherapy with activity against a wide range of human malignancies. Antifolates have been been used to treat some nonmalignant disorders including psoriasis, rheumatoid arthritis, as well as bacterial and parasitic infections. This class of agents represents the best-characterized and most versatile of all chemotherapeutic drugs in the current clinical use (1). 

MTX is a potent inhibitor of dihydrofolate reductase (DHFR) which inhibits the precursors of nucleic acids and cell proliferation (2). However, it has a short plasma half-life (1.5–3.5 h), short tumor exposure time, and consequently an impaired therapeutic efficacy (3). In contrast, MTX accumulates in normal tissues especially liver, kidneys, and intestine producing severe toxicities like ulcerative colitis, hepato, and nephrotoxicity (3, 4). In addition to these non-selective toxicities, MTX has a high occurrence of drug resistance which limits its effectiveness. It has been reported that 30% of remission failures in the treatment of ALL are due to MTX resistance (5). 

One of the earliest reports on advantages of MTX polymer conjugation was overcoming drug resistance that can be due to bypassing efflux pumps effective on free drug as well as blocking over-expression of these pumps (6, 7). Another advantage of conjugation with macromolecules is the passive accumulation of conjugates in solid tumors because of the enhanced permeability and retention (EPR) effect due to leaky tumor vasculature and poorly developed lymphatic drainage (8, 9). This accumulation reduces systemic toxicity by reducing damage to non-cancerous organs (10). The accumulation may account for a several fold higher maximum tolerated dose of a conjugate compared to the free drug (11). In addition, the EPR effect is amplified by the conjugate cytotoxicity, which increases the average drug concentration in the tumor and enhances the conjugate efficacy (12). For the above mentioned reasons, the researchers have done much effort to conjugate the drug with natural and synthetic polymers including dextran, gelatin, polyaspartide and human serum albumin (13-16).

In recent years, a lot of attention has been focused on polyethylene glycol (PEG) and its derivatives for drug conjugation (PEGylation) which is based on the unique characteristics of polymer for improving solubilization, stabilization, and passive targeting of anticancer drugs. Many of these drugs have been conjugated by PEG and are being under clinical phases I and II. PEG-camptothecin, PEG-irinotecan, PEG-docetaxel and PEG-paclitaxel are the pegylation products of these anticancer drugs under development (17-21). The overall goals of these conjugations are to increase solubility and blood circulation time, in order to decrease side effects and overcome the drug resistance of the tumor cells. Pegylation has been also successfully tried for efficient delivery of other anticancer drugs including Doxorubicin, Epirubicin, Cytosine arabinoside and Gemcitabine (22-25). In all cases, the conjugation led to more effective compounds against tumor cells relative to native drugs. These observations approve the pegylation as an invaluable tool for more efficient drug delivery of chemotherapeutic agents. In addition, other pegylated nanostructures have been extensively studied for MTX delivery to tumor cells like pegylated chitosan coated magnetic nanoparticles and pegylated polyethylene imine for rheumatoid arthritis and pegylation plays a significant role in *in-vitro* stability and *in-vivo* efficiency of the systems (26, 27). 

 In our previous work, we reported the synthesis and characterization of pegylated conjugates of MTX with different molecular weights of PEG including 750, 5000, and 35000 D (28). Water solubility, partition coefficient, and chemical stability of conjugates were determined and MTX-PEG5000 was chosen as suitable compound for further investigations. The current work aims to study pharmacokinetic and biodistribution of MTX-PEG5000 after IV administration to mice.

## Experimental

*Materials*

MTX-PEG5000 was prepared in-house. MTX was kindly gifted by HEUMANN PCS Company (Feucht, Germany). Heparin 5000IU ampoules were supplied by Daroupakhsh Company (Iran). Cremophor RH 40 was purchased from Fluka (U.S.A) and t-buthyl-ethyl ether was obtained from Merck KGaA Company (Darmstadt, Germany). Dialysis bag with cut off 12KD was supplied by Millipore® (U.S.A). All other reagents were at least analytical grade and the solvents were HPLC grade. 

*Methods*

*Preparation of MTX and MTX-PEG5000 plasma samples and standards*

A portion of 500 μL acetonitrile was added to a mixture of 225 μL plasma and 25 μL MTX or MTX-PEG5000 solution (100 µg/mL) and vortexed. Precipitated proteins were separated by centrifuging the mixture at 12000 rpm for 5 min. The supernatant was evaporated by a Multi-Evaporator (VLM EC 1, Italy) at 50 °C using nitrogen gas. The residue was dissolved in 150 μL mobile phase and injected to HPLC. The standards were prepared in triplicate at eight concentrations ranging from 0.25 to 100 µg/mL for MTX and from 1 to 1000 µg/mL for MTX-PEG5000. 

*Preparation of MTX and MTX-PEG5000 tissue samples and standards*

For preparation of MTX tissue samples, one volume of tissue homogenate in normal saline was mixed with two volume acetonitrile and centrifuged in 12000 rpm for 5 min. The supernatant was removed and evaporated to dryness under nitrogen gas. The residue was reconstituted in 250 μL mobile phase and was injected to HPLC. For preparation of MTX-PEG5000 tissue samples, one volume of tissue homogenate in normal saline was mixed with two volumes of dichloromethane and shaked for 15 min. After centrifugation (5000 rpm), the lower organic phase was aspirated and dehydrated by Na_2_SO4 and after dissolving in 200 μL mobile phase, was injected to HPLC. The standard solutions were prepared in triplicate at concentration levels ranging from of 1 to 50 µg/mL for MTX and 2.5 to 500 µg/mL for MTX-PEG5000.

*HPLC method for analysis of MTX and MTX-PEG5000 plasma samples*

A Knauer HPLC system consisted of a wellchrom pump k-1001, equipped with k-2701, DAD k-2700 detector was used. Chromatography was performed on a PerfectSilTarget® 100 HPLC Column (C8, 150 × 4.6 mm, 5 µm). For analysis of MTX, mobile phase consisted of phosphate-citrate buffer (pH 5): acetonitrile (88:12, v/v) and was delivered isocratically at a flow rate of 1 mL/min. 

The column eluent was monitored using UV detector at the wavelength of 302 nm. For analysis of MTX-PEG5000, the mobile phase consisted of phosphate-citrate buffer (pH 5): acetonitrile (55:45, v/v) and was delivered isocratically at a flow rate of 0.5 mL/min. The column eluent was monitored using UV detector at the wavelength of 342 nm. 

The precision and accuracy of the method were examined by adding known amounts of MTX (1, 10 and 100 µg mL-1) and MTX-PEG5000 (5, 50 and 500 µg/mL) to pool plasma (quality control samples). For intra-day precision and accuracy, five replicate quality control samples at each concentration were assayed on the same day. The inter-day precision and accuracy were evaluated on three different days. The method specificity was assessed for drug separation from its major metabolite, 7- hydroxyl methotrexate. 

*HPLC method for analysis of MTX and MTX-PEG5000 tissue samples*

The same HPLC instrument was utilized for analysis of MTX and MTX-PEG tissue samples. The precision and accuracy of the method were examined by adding known amounts of MTX (0. 25, 2.5 and 10 µg/mL) and MTX-PEG5000 (5, 50 and 500 µg/mL) to pool tissue (quality control) samples. The other steps were performed as plasma samples.

*Study of MTX-PEG5000 hydrolysis by plasma and liver homogenates*

The stock solutions of MTX-PEG5000 were prepared by dissolving appropriate amounts of the conjugate in acetonitrile in order to obtain a concentration of 100 µg/mL. All stock solutions were kept in screw-capped vials at 4 °C. The reaction was initiated by adding 25 μL of stock solutions to 225 μL of preheated plasma or 450 μL liver homogenate samples in screw-capped vials at 37.5 °C. 

The solutions were kept in a water bath at 37.5 °C and at appropriate time intervals (6, 12, 24, 30, 36, 48, 60, 72, 96, and 144 h for plasma samples and 12, 24, 30, 36, 48, 60, 72, 96, and 144 h for liver homogenate samples), 20 μL aliquots were taken and frozen in -20 °C. 

Finally, the samples were prepared as mentioned and analyzed by HPLC. All experiments were repeated three times under the same conditions. Pseudo-first order rate constants for the hydrolysis of the conjugate were determined from the slopes of the linear plots of the logarithm of remaining ester against time.

*Study of MTX-PEG5000 Protein binding*

To perform the study, equilibrium dialysis method was employed using a protein binding bath (ERWEKA, Germany) (30-32). A cellulose acetate dialysis membrane with cut off 12KD was used between two phases. Different concentrations of the conjugate in plasma including 50, 100, 250, and 500 µg/mL were prepared and 2 mL of each concentration was placed in receiver side and allowed to equilibrate with the same volume of Krebs buffer at 37 °C. After 5 h, the equilibrium was reached and the conjugate concentration was assayed in both buffer and plasma sides using HPLC method.

*Preparation of MTX and MTX-PEG5000 IV solutions*

MTX-PEG5000 was synthesized, purified, and characterized in our laboratories (28). For preparation of MTX solution, the drug was firstly suspended in sterile saline solution and then a clear solution was obtained by adding concentrated NaOH solution to a final pH of 8. Then, the solution was proportionally diluted to obtain a 10 mg/mL MTX solution. For preparation of 10 mg/mL MTX-PEG5000 solution, different cosolvents and nonionic surfactants including ethanol, propylene glycol, PEG600, DMSO, Tween 20, Tween 80, and Cremophore RH-40 were tested and 10% Cremophore RH-40 was used as resulted to a clear and stable formulation. 

*Pharmacokinetics and biodistribution study of MTX and MTX-PEG5000*

IV bolous injections of MTX and MTX-PEG5000 (70 mg/kg) were administered to mice through marginal veins by insulin syringe (gauge 27). At least three mice were sacrificed at each time point. The animals (n = 200) at dosing were in the range of 2-4 weeks of age and their mean weight was 20 ± 5 g. The blood samples were collected at 5, 10, 15, 30, 45, 60, 90, and 120 min intervals for MTX and for at 5, 10, 15, 30, 45, 60, 90, 120, 180, 240, 300, and 360 min intervals for MTX-PEG5000. At each time point, the blood samples were collected from at least three mice and after centrifuging (10000 rpm, for 5 min), the plasma samples were separated and immediately frozen at -20 °C. Then, the mice were cervically dislocated and their organs including heart, liver, spleen, kidney, lung, brain, and small intestine were removed and after washing with normal saline, were dried by facial tissue and exactly weighed. Whole removed organs and 0.25 to 0.5 g of liver and small intestine were minced and homogenized in a certain volume of normal saline using homogenizer IKA basic T25 (Germany). The organs were collected from MTX injected animals (10, 60 and 120 min for all organs and 5, 10, 30, 60 and 120 for liver tissue) and at 2, 6, 12, 24, and 48 h for mice administered by MTX-PEG5000.

The logarithm of plasma concentrations of MTX and MTX-PEG5000 were plotted *vs.* time. Both MTX and MTX-PEG5000 showed two-compartmental pharmacokinetic model and therefore, the elimination and distribution rate constants (β and α) were obtained from terminal elimination and residual line, respectively. The pharmacokinetic parameters were obtained by fitting data to two-compartmental model equation (C = Ae^-αt^ + Be^-βt^) and noncompartmental model using Excel 2010 (33).

## Results

*Preparation of MTX plasma and tissue samples*

The use of different ratios of acetonitrile showed that the ratio 2:1 of solvent to plasma could lead to 95% mean recovery in all concentrations. The chromatograms were neater than acid precipitation method and pressure increase was lower. For tissue preparations, the same method was employed but recovery was 70%. 

**Table 1:** Method validation parameters of HPLC method for MTX and MTX-PEG5000 quantification in plasma

	**C (mg/mL)**	**intra-day (CV%)**	**inter-day (CV%)**	**Accuracy (%)**
	0.001	4.04	4.18	95.10
MTX	0.01	2.15	2.97	99.14
	0.1	3.53	4.12	102.32
	0.00025	4.66	4.86	92.7
MTX-PEG5000	0.0025	0.89	1.31	104.05
	0.01	3.78	3.52	96.8

**Table 2:** Method validation parameters of HPLC method for MTX and MTX-PEG5000 quantification in tissue homogenate

	**C (mg/mL)**	**intra-day (CV%)**	**inter-day (CV%)**	**Accuracy (%)**
	0.005	4.66	4.86	97.14
MTX	0.05	0.89	1.31	91.28
	0.5	3.78	3.52	101.93
	0.005	4.10	4.58	104.38
MTX-PEG5000	0.05	3.85	3.33	100.15
	0.5	5.25	4.63	93.70

**Table 3:** MTX-PEG5000 stability half-life in plasma and liver homogenate in 37 °C. The data are the average of three replicates

**T** **1/2 ** **(h)**		
**Buffer pH 7.4**	**Plasma**	**Liver homogenate**
3528 ± 53	158 ± 5	23 ± 1

**Table 4:** Protein binding of different concentrations of MTX-PEG5000 in plasma determined by equilibrium dialysis method

**C (mg/mL)**	**C (plasma)**	**C (buffer)**	**Protein binding (%)**
0.05	0.0366 ± 0.0011	0.0016 ± 0.0001	95.8 ± 3.8
0.1	0.0684 ± 0.0014	0.0033 ± 0.0001	95.4 ± 3.4
0.25	0.1512 ± 0.0068	0.0069 ± 0.0003	95.6 ± 4.3
0.5	0.2730 ± 0.0136	0.0170 ± 0.0006	94.2 ± 3.3

**Table 5:** Solubility MTX-PEG5000 in different cosolvents and surfactants

	**max percent used**	**Solubility (mg/mL)**
propyleneglycol	50%	1.5 ± 0.1
PEG 600	40%	2.0 ± 0.2
Ethanol	50%	5.5 ± 0.3
DMSO	20%	2.5 ± 0.2
Tween 20	30%	20 ± 1.5
Tween 80	30%	30 ± 1.5
Cremophore RH-40	25%	30 ± 1.5

**Table 6:** Pharmacokinetic parameters of MTX and MTX-PEG5000 after iv injection to mice (n = 3).

	**MTX**	**MTX-PEG 5000**
T1/2a (min)	2.445 ± 0.549	9.164 ± 1.927
T1/2b (min)	24.328 ± 0.657	88.436 ± 6.710
MRT (min)	9.304 ± 0.409	98.217 ± 7.069
AUC (mg.mL-1.min)	2.642 ± 0.190	12.327 ± 0.914
AUMC (mg.mL-.min2)	24.63 ± 2.84	1213.027 ± 158.425
CLs (mL/min)	22.401 ± 5.465	5.994 ± 0.448
Vc (mL)	94.025 ± 6.814	237.338 ± 30.685
K10 (min ) -1	0.236 ± 0.043	0.026 ± 0.004
K12 (min ) -1	0.050 ± 0.020	0.036 ± 0.011
K21 (min ) -1	0.036 ± 0.002	0.024 ± 0.004

**Figure 1:**
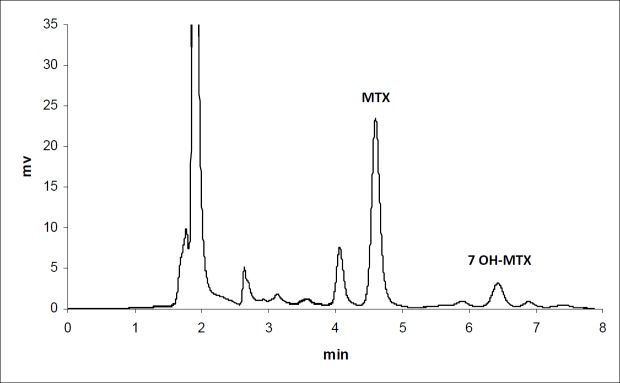
Chromatogram of MTX in plasma 30 min after iv injection to mice (n = 3)

**Figure 2:**
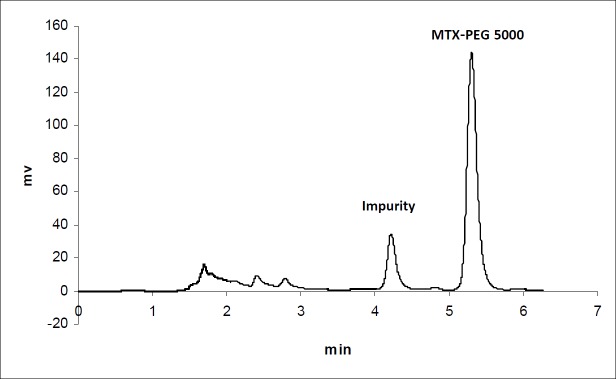
Chromatogram of MTX-PEG5000 in plasma 30 min after iv injection to mice

**Figure 3:**
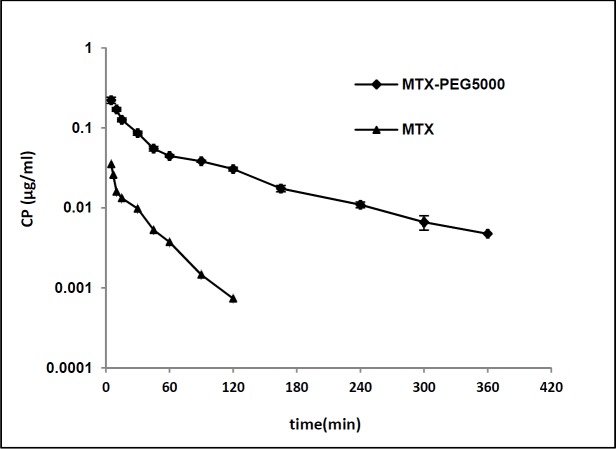
Plasma concentration-time profile of MTX and MTX-PEG5000 after iv injection to mice (dose 70 mg/kg for both, n = 3, semi-log scale).

**Figure 4.**
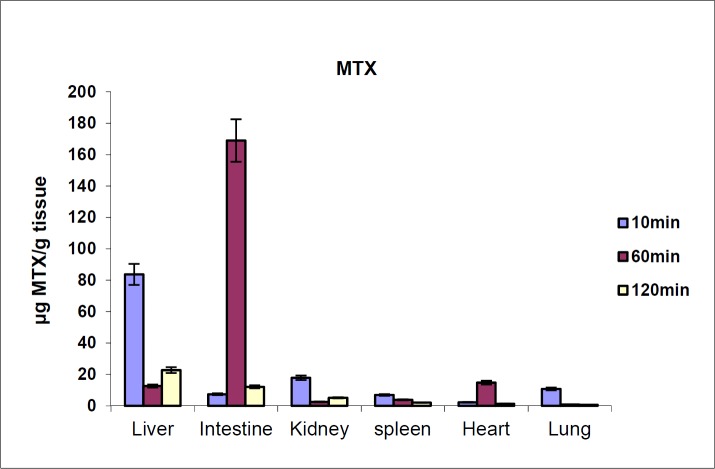
MTX tissue distribution in mice in different times after iv injection to mice (dose 70 mg/kg, n = 3)

**Figure 5:**
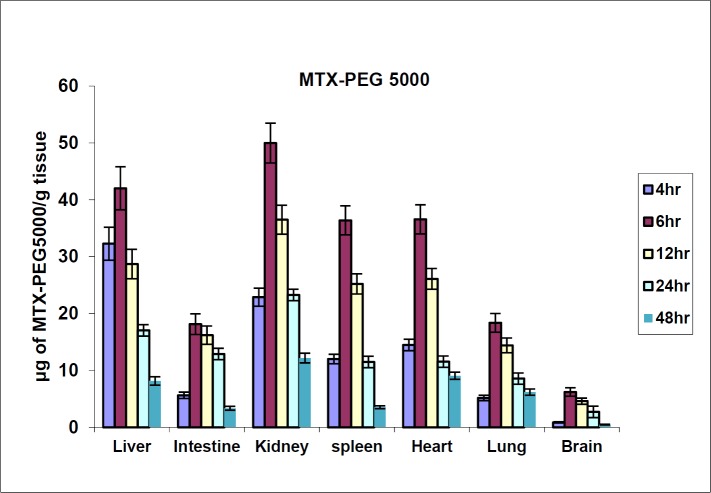
MTX-PEG5000 tissue distribution in mice in different times after iv injection (dose 70 mg/kg, n = 3)

*Preparation of MTX-PEG5000 plasma and tissue samples*

For MTX-PEG5000, a recovery of 92-100% was obtained by protein precipitation method using acetonitrile. Tissue sample preparation by protein precipitation produced 100% recovery but many interfering peaks were observed in chromatograms. Extraction of conjugate by dichloromethane led to clean samples for injection to HPLC with average of 85% recovery. 

*HPLC method for analysis of MTX and MTX-PEG5000 plasma samples*

The calibration curves for the determination of MTX and MTX-PEG5000 in plasma were linear over the concentration ranges of 0.1-100 and 0.25-500 μg/mL, respectively. For each calibration curve, the intercept was not statistically different from zero. The correlation coefficients (r) for calibration curves were equal to or better than 0.999. For each point of calibration standards, the concentrations were recalculated from the equation of the linear regression curves. The limits of quantification (LOQ) were 0.10 and 0.25 μg/mL for MTX and MTX-PEG5000, respectively. Intra and inter-day coefficients of variation for studied concentrations of MTX and MTX-PEG5000 were less than 5% (Table 1). The method accuracies were in the range of 100 ± 5% for MTX and 100 ± 10% for MTX-PEG5000 and dependence on concentration was negligible. Figures 1 and 2 show the chromatograms of MTX and MTX-PEG5000 after preparation of plasma samples of mice 30 min after IV injections.

*HPLC method for analysis of MTX and MTX-PEG5000 tissue samples*

For tissue samples, the linearity ranges were 0.25-100 μg/mL and 2.5-500 μg/mL for MTX and MTX-PEG5000, respectively (R ≥ 0.999) and intercepts were not significantly different from zero (*p* > 0.05). The limits of quantification (LOQ) were 0.25 and 2.5 μg/mL for MTX and MTX-PEG5000, respectively. Intra and inter-day coefficients of variation for all concentrations of MTX and MTX-PEG5000 were less than 5% (Table 2). The method accuracies were in the range of 100 ± 10% for both MTX and MTX-PEG5000. 

*MTX-PEG stability study in plasma and liver homogenate at 37 °C*

*In-vitro* plasma stability study showed a pseudo-first order degradation of MTX-PEG5000 with a half-life of 158 h (over one week) (Table 3). The results obtained from liver homogenate showed a two-segmental pseudo-first order release with a very fast initial dissociation (releasing 50% MTX in first 24 h) and a slow secondary release stage (50% in longer than 120 h ) (Figure 3).

*Study of MTX-PEG5000 protein binding*

The conjugate showed an average of 95% protein binding in a wide range of concentrations from 50 to 500 µg/mL (Table 4). The results showed that MTX-PEG5000 highly binds to plasma proteins (> 90%) and is less likely present as a free compound in plasma or blood. For MTX, The protein binding was reported in the literature as 30-50% (34, 35). 

*Preparation of MTX and MTX-PEG5000 solutions for IV injection*

MTX was solubilized in water by adjusting pH to 8. In contrast, low solubility of MTX-PEG5000 was not affected by pH of the solution. In order to solubilize the conjugate in water, different surfactants were examined, amongst them nonionic surfactants Tween 80 and Cremophore RH-40 resulted in clear solutions at acceptable concentration ranges (Table 5). However, the solutions obtained using Tween 80 were not physically stable and crystallization was observed after 24 h. The solutions of the conjugate obtained by 30% w/w of Cremophore RH-40 were clear and stable at conjugate concentrations up to 30 mg/mL. However, the resulting highly viscose solutions were not suitable for iv injection and Cremophore/water 10:90% w/w was chosen to make 10 mg/mL of MTX-PEG5000 solutions for iv injection to mice.

*MTX and MTX-PEG5000 pharmacokinetic and biodistribution study*

Figure 3 shows plasma concentration-time profile of MTX and MTX-PEG5000 after iv injection to the mice in logarithmic scale. The pharmacokinetic parameters were calculated fitting data to two-compartmental and noncompartmental models. According to the results, both the drug and its conjugate showed very good compliance with two-compartmental pharmacokinetic model having good linearities for extrapolated and residual lines (R^2 ^> 0.97). Also, the noncompartmental model was employed to obtain mean residence time (MRT). The pharmacokinetic parameters of MTX and MTX-PEG5000 are given in Table 6. The conjugate form shows significantly longer distribution and elimination half-lives than MTX (9.164 *vs.* 2.445 min (*p* < 0.01) and 88.436 *vs.* 24.328 min (*p *< 0.0001), respectively). MRT increased from 9.304 min for MTX to 98.217 min for MTX-PEG5000 (*p *< 0.0001). Systemic clearance (CLs) decreased 3.7 times from 22.401 mL/min for MTX to 5.994 mL/min for MTX-PEG5000 (*p *< 0.01) and AUC increased 4.7 times from 2.642 mg.min/mL for MTX to 12.327 mg.min/mL for MTX-PEG5000 (*p *< 0.0001). 

The biodistribution results of MTX at defined intervals after injection (10, 30 and 60 min) are shown in Figure 4. The results showed that MTX accumulated in liver immediately after its distribution phase (10 min) and then redistributed to small intestine (60 min). Also, no detectable amount of drug was found in the brain. In contrast, distribution results of MTX-PEG5000 showed that the conjugate did not highly accumulate in different organs specially liver and intestine (Figure 5). Also, the conjugate was measurable in tissues up-to 48 h after injection (compared to 2 h for MTX).

## Discussion

*Preparation of MTX and MTX-PEG5000 solutions for IV injection*

MTX is more soluble in alkaline aqueous solutions (58.17 mg/mL in pH8 *vs.* 0.08 mg/mL in pH4) which is attributed to the ionization of α and γ carboxylic groups specially α ones (pka 3.5) (25). After pegylation, this group is esterified and cannot be ionized. Therefore, the conjugate is more lipophilic in character (logP 4.3) (28). But nonionic surfactants like Tween 80 and Cremophor RH-40 can solubilize it by micelle forming mechanisms. 

*Preparation of MTX plasma and tissue samples*

MTX is ionized and soluble in mild alkaline solutions. A 50% portion of the drug binds to plasma proteins. Acetonitrile can easily dissociate MTX from precipitated plasma proteins, whilst in more complex matrices like tissue homogenates, the solvent is not as efficient as in plasma in the extraction of the drug. Thus, the recovery of the extraction of MTX from plasma is higher than that from tissue homogenates (95% compared to 70%, respectively). 

*Preparation of MTX-PEG5000 plasma and tissue samples*

As mentioned before, MTX-PEG5000 is a lipophilic conjugate with low solubility in mild alkaline solutions including plasma. Moreover, it is highly bound to plasma proteins (>95%). Therefore, the relatively nonpolar immiscible solvents like dichloromethane can extract the compound and relatively polar miscible solvents like acetonitrile can recover it by precipitation of plasma proteins.

*HPLC method validation for analysis of MTX and MTX-PEG5000 plasma samples*

The method was sensitive enough for MTX quantification as necessary in pharmacokinetic studies (LOQ 0.1 μg/mL). The method showed to be accurate and precise (inaccuracy < ±5% and CV < ±5%) over a wide range of the drug concentrations. Similar results were obtained for MTX-PEG5000, although the method was not as sensitive as for MTX (LOQ 0.25 μg/mL) and inaccuracy was less than ±10%. This is likely due to lower recovery and less molar absorptivity of conjugate compared to MTX. 

*HPLC method validation for analysis of MTX and MTX-PEG5000 tissue samples*

More complex matrix of tissue samples led to less sensitive results than plasma samples (LOQ 0.25 and 2.5 μg/mL for MTX and MTX-PEG5000, respectively). However, the method sensitivities were high enough to quantitate the drug and its conjugate in tissue homogenates at different time points up-to 48 h. As for plasma, the methods proposed for tissue analysis were accurate and precise (inaccuracy < ±10% and CV < ±5%) for both MTX and MTX-PEG5000.

*MTX-PEG stability study in plasma and liver homogenate in 37 °C*

Plasma stability of drug-polymer conjugates is very important. Some polymer conjugates are designed to release the drug gradually. This strategy is suitable either for short acting drugs like ketoprofen (35) or potent and toxic drugs with burst release like warfarin (36). In the case of pegylated anticancer drugs like PEG-Campthotecine, stable linkages can lead to passive targeting (EPR effect) (39). Our results demonstrated that the conjugate could maintain the drug in the systemic circulation for longer time. This time seems to be enough for the conjugate to be entrapped by tumor tissues (EPR effect) and internalized by endocytosis. Thereafter, it may degrade and release the drug in the site of action by endosomal enzymes.

*Study of MTX-PEG5000 protein binding*

Protein binding is one of the most important factors influencing drug kinetics and distribution. Our previous study revealed the low solubility and high partition coefficient of MTX-PEG5000 in pH values above 7 (28, 29). Therefore, it is suggested that the low solubility of the conjugate in plasma matrix, drives the conjugate to bind to plasma proteins. In contrast, MTX has a protein binding of 30-50% due to ionization of carboxylic groups in plasma and shows higher solubility compared to MTX-PEG5000 (36, 37). This difference can affect elimination rate of the conjugate and its distribution to the organs as the conjugate will probably stay in blood circulation which can increase the accumulation in tumor tissues via well-known enhanced permeability retention (EPR) effect.

*MTX and MTX-PEG5000 pharmacokinetic and biodistribution study*

MTX is an ionisable polar drug in relatively alkaline pH of plasma (logP = -1.4) and hence, has short plasma circulating time. As shown in our first paper (29), MTX-PEG5000 is a lipophilic conjugate (logP = 4.3) which is highly protein bound (about 95%). Therefore, after iv injection, it is reasonable to observe a long elimination half-life for conjugate compared to free drug. The similar results have been reported by other researchers for PEG-Campthotecin, PEG-Gemcitabine (25, 38). In peg-Campthotecin (Prothecan®), the link of drug to PEG40000 has caused to a long plasma circulation compared to free drug (T_1/2_ β 77 h *vs.* 22 h) as a weekly injection is sufficient and more efficient than once daily injection for a week. Regarding PEG-Gemcitabine, very similar results to our research were observed. Conjugation to PEG5000 led to longer T_1/2_ α (T_1/2_ β of 7.13 *vs.* 0.99 h), longer T_1/2_ β (32.23 *vs.* 11.23 h), higher AUC (382.87 vs. 118.55 μg min/mL), and lower clearance (2.11 *vs.* 0.654 mL/min) for Gemcitabin-PEG5000 compared to Gemcitabine. These results indicate that drug pegylation with a PEG5000 can modify the pharmacokinetic of a hydrophilic drug like gemcitabine similar to MTX. For a good survey on pharmacokinetic consequences of pegylation, the readers can refer to Hamidi *et al.*

(39). 

One of the most important consequences of pegylation relates to the effect of pegylation on drugs biodistribution. Unfortunately, there is no certain study on pegylated small molecule drugs in this regard and the researchers have focused on pegylated nanocarriers instead of drug itself. In current study, we compared biodistribution of MTX and MTX-PEG5000 after iv injection to mice. MTX showed a high and fast accumulation in liver that can be responsible to its limiting liver toxicity. The drug redistribution to small intestine is in good agreement with the results reported previously and can be the reason of acute gastrointestinal adverse effects of the drug (41). 

It is well known that blood brain barrier is not permeable to MTX and hence, intrathecal injection is the only way of drug delivery to brain tumors which causes serious reactions like acute arachnoiditis (42). In contrast to MTX, the conjugate did not show fast accumulation in liver and small intestine which may result in lower incidence of acute liver and gastrointestinal toxicities. Also, the conjugate had a long tissue residence time which can be due to its lipophilic nature and high molecular weight which may reduce clearance of the drug from the organs. Another interesting result was that MTX-PEG5000 is quantified in the brain that means it can permeate to BBB and provides the possibility of brain tumor drug delivery without using intrathecal injections. PEG5000 can act as a brain vector for MTX and mechanisms like endocytosis facilitates the drug transfer to the brain (43). 

This finding can also be explained by lipophilic nature of pegylated drug in comparison to hydrophilic nature of the native drug. This explanation is in good agreement with that found for the new MTX analogs entering clinical phases. They are lipid soluble antifolates like trimetrexate and pyritrexim which the ionizable carboxylic acid groups have been replaced with nonionizable aromatic groups. 

This modification has caused to longer plasma elimination half-life and breaking the tumor cell resistance (1). Also, some lipophilic esters of MTX have been synthesized and shown to be more effective than native drug against drug resistant tumor cells (44). Furthermore, some other mechanisms of overcoming tumor resistance can be supposed with these conjugates including p-gp efflux transporters inhibition (45). All of these features can be expected by MTX-PEG5000 because of its higher lipophilicity and its possibility to entering the cells by extra mechanisms like endocytosis.

## Conclusion

Polymer conjugation especially pegylation has recently become an excellent strategy for drug delivery of anticancer drugs. MTX as a common drug in majority of chemotherapy regimens has been conjugated with many natural and synthetic polymers. In the current study, the pharmacokinetic and tissue distribution of MTX-PEG5000 were studied. The results of this study showed that conjugation could significantly increase drug circulation time in blood. This can be due to high molecular weight of the polymer and also high protein binding of the conjugate which may prevent rapid glomerular filtration. The stability studies showed that the conjugate is stable enough in plasma (T_1/2_ = one week) but breaks down in presence of liver homogenate (T_1/2_ = 1 day). These results are promising to passive targeting of conjugate (EPR effect) and can reduce drug side effects due to slow release of MTX before reaching to the rich enzymatic media of the tumor cells. On the other hand, the pharmacokinetic studies showed a significantly longer blood circulation for conjugate compared to the parent drug (10 times longer mean residence time, MRT) and biodistribution studies showed slow distribution of conjugate in different organs without acute accumulation in liver, kidneys, and small intestine. Also, MTX-PEG5000 showed the ability to pass the BBB promising the possibility of drug delivery to brain tumors without dangers of intrathecal injection. In conclusion, the results obtained in the present study indicate that the esterification of MTX with mPEG5000 can be considered as an efficient strategy to increase efficacy and to decrease acute toxicity of the drug. 
